# Incidental Diagnostic and Treatment of a Suppurative Appendicitis at Colonoscopy

**DOI:** 10.1155/2012/523708

**Published:** 2012-06-17

**Authors:** Mohammed Amine Benatta

**Affiliations:** Gastroenterology Department, Military Universitary Hospital of Oran, 156 Khemisti, Oran, Algeria

## Abstract

We herein report a patient with acute appendicitis who was diagnosed by colonoscopy: a 54-year-old woman with normal physical examination and laboratory results within normal limits. The case had shown an erythematous and edematous bulging polypoid lesion with purulent discharging from appendiceal orifice into the cecum. At colonoscopy, appendix appeared as an erythematous and edematous bulging polypoid lesion with purulent discharging from appendiceal orifice into the coecum. Surgeons exclude any emergency. Two weeks later, a colonoscopic second look was performed, and the same bulging polypoid lesion was found but without spontaneous purulent discharging. Appendiceal endoscopic pus drainage was then performed.

## 1. Case Report

**Figure 1:**
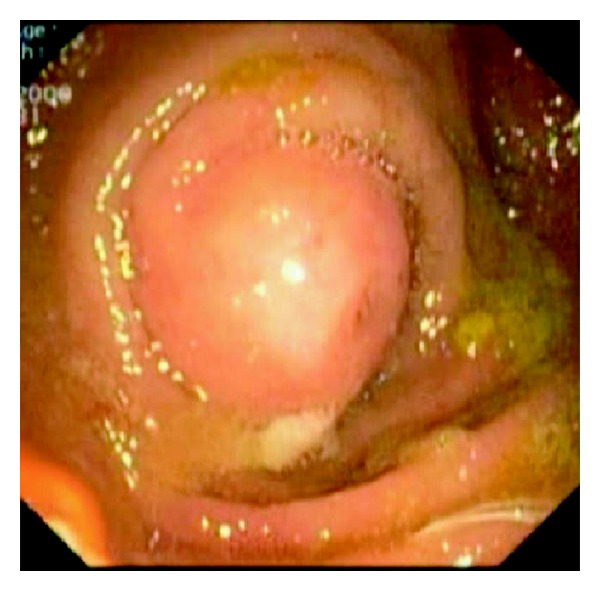
Bulging polypoid lesion into the cecal lumen.

**Figure 2:**
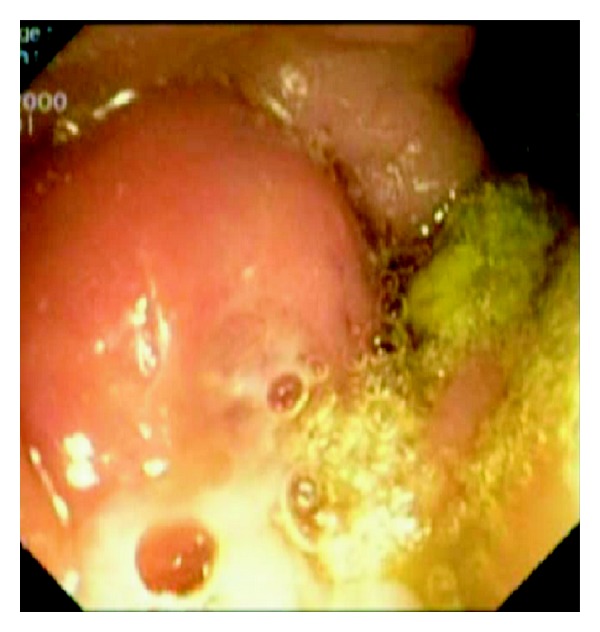
Purulent discharging from the appendiceal orifice.

**Figure 3:**
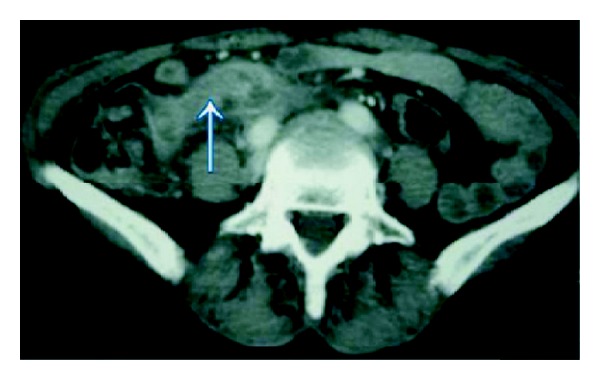
Tumefied appendice with local infiltra.

**Figure 4:**
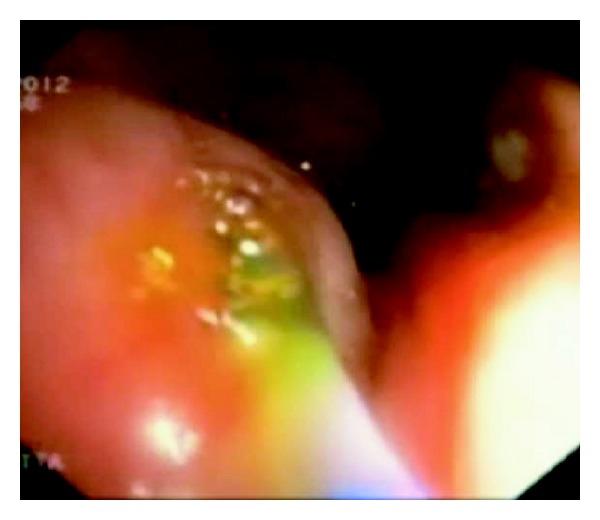
Appendiceal orifice irrigated using a catheter and pus flowing.

A 54-year-old woman was referred for upper endoscopy for dysphagia. She had a history of esophagectomy with coloplasty for stenosis due to corrosive injuries 5 years ago. Physical examination was normal and laboratory results were within normal limits. Upper endoscopy showed an anastomotic esophageal-colonic stricture with a big diverticulum on the coloplasty. At this moment, our patient receives antibiotherapy for urinary infection. Three days later, there was no diverticulum and the colocolic anastomosis was without abnormalities at colonoscopy, but an erythematous and edematous bulging polypoid lesion (Figure 1) with purulent discharging from appendiceal orifice into the coecum (Figure 2) appeared. These endoscopic features were strongly suggestive of appendicitis. Antibiotherapy was stopped and laboratory results showed mild leucocytosis. The abdominal computed tomography findings were a tumefied appendix with local infiltration and a blade liquid (Figure 3). Therefore, the case was referred to surgery. Surgeons conclude that there was no emergency given the normality of physical examination and laboratory results being within normal limits. Two weeks later, we performed a colonoscopic second look, the same feature of bulging polypoid lesion into the cecal lumen was found but without purulent discharging this time. To drain the pus, the appendiceal orifice was intubated and abundantly irrigated with saline solution using ERCP endoscopic retrograde cholangio-pancreatography catheter resulting in residual pus flowing (Figure 4). Three months later, our patient remains asymptomatic.

## 2. Discussion

It is an atypical case of acute appendicitis diagnosed and treated at colonoscopy, while initial presentation was without any suggestive symptoms. The diagnosis of acute appendicitis is frequently based on clinical criteria and often supported by results from simple blood tests. Ultrasonography and/or computed tomography are the main appropriate imaging modalities [1]. For delayed or atypical presentations, colonoscopy is also reported as a diagnostic examination for acute appendicitis [1–3]. Endoscopic treatment was reported in only two cases [4, 5]. One case of asymptomatic patient in whom appendicitis was both diagnosed and treated during colonoscopy has been reported before [4]. One case of patient with a prohibitive surgical risk treated by colonoscopy has been reported [5]. Our patient is one of these rare patients among whom acute appendicitis was incidentally diagnosed at colonoscopy and also treated endoscopically by appendiceal pus drainage.
